# From laptops to supercomputers: a single highly scalable code base for spiking neuronal network simulations

**DOI:** 10.1186/1471-2202-14-S1-P163

**Published:** 2013-07-08

**Authors:** Susanne Kunkel, Maximilian Schmidt, Jochen M Eppler, Hans E Plesser, Jun Igarashi, Gen Masumoto, Tomoki Fukai, Shin Ishii, Abigail Morrison, Markus Diesmann, Moritz Helias

**Affiliations:** 1 Simulation Laboratory Neuroscience - Bernstein Facility Simulation and Database Technology, Institute for Advanced Simulation, Jülich Aachen Research Alliance, Jülich Research Centre, Germany; 2 Institute of Neuroscience and Medicine (INM-6) and Institute for Advanced Simulation (IAS-6), Jülich Research Centre and JARA, Germany; 3 Bernstein Center Freiburg, Albert-Ludwig University of Freiburg, Germany; 4 Department of Mathematical Sciences and Technology, Norwegian University of Life Sciences, Aas, Norway; 5 Laboratory for Neural Circuit Theory, RIKEN Brain Science Institute, Wako, Japan; 6 High-Performance Computing Team, RIKEN Computational Science Research Program, Kobe, Japan; 7 Integrated Systems Biology Laboratory, Department of Systems Science, Graduate School of Informatics, Kyoto University, Japan; 8 Institute of Cognitive Neuroscience, Faculty of Psychology, Ruhr-University Bochum, Germany; 9 Medical Faculty, RWTH University, Aachen, Germany

## 

Over the last couple of years, supercomputers such as the Blue Gene/Q system JUQUEEN in Jülich and the K computer in Kobe have become available for neuroscience research. These massively parallel systems open the field for a new class of scientific questions as they provide the resources to represent and simulate brain-scale networks, but they also confront the developers of simulation software with a new class of problems. Initial tests with our neuronal network simulator NEST [1] on JUGENE (the predecessor of JUQUEEN) revealed that in order to exploit the memory capacities of such machines, we needed to improve the parallelization of the fundamental data structures. To address this, we developed an analytical framework [2], which serves as a guideline for a systematic and iterative restructuring of the simulation kernel. In December 2012, the 3^rd ^generation technology was released with NEST 2.2, which enables simulations of 10^8 ^neurons and 10,000 synapses per neuron on the K computer [3].

Even though the redesign of the fundamental data structures of NEST is driven by the demand for simulations of interacting brain areas, we do not aim at solutions tailored to a specific brain-scale model or computing architecture. Our goal is to maintain a single highly scalable code base that meets the requirements of such simulations whilst still performing well on modestly dimensioned lab clusters and even laptops.

Here, we introduce the 4^th ^generation simulation kernel and describe the development workflow that yielded the following three major improvements: the self-collapsing connection infrastructure, which takes up significantly less memory in the case of few local targets, the compacted node infrastructure, which causes only negligible constant serial memory overhead, and the reduced memory usage of synapse objects, which does not affect the precision of synaptic state variables. The improved code does not compromise on the general usability of NEST and will be merged into the common code base to be released with NEST 2.4. We show that with the 4g technology it will be possible to simulate networks of 10^9 ^neurons and 10,000 synapses per neuron on the K computer.
